# Alexithymia and Psychopathological Manifestations Centered on the Body: Somatization and Self-Harm

**DOI:** 10.3390/jcm11082220

**Published:** 2022-04-15

**Authors:** Michela Gatta, Caterina Angelico, Francesca Rigoni, Alessia Raffagnato, Marina Miscioscia

**Affiliations:** 1 Child and Adolescent Neuropsychiatric Unit, Department of Women’s and Children’s Health, University Hospital of Padua, 35128 Padua, Italy; michela.gatta@unipd.it (M.G.); francesca.rigoni.4@studenti.unipd.it (F.R.); alessia.raffagnato@unipd.it (A.R.); 2 Department of Developmental Psychology and Socialisation, University of Padua, 35131 Padua, Italy

**Keywords:** alexithymia, somatization, emotional dysregulation, non-suicidal self-injury, self-harming, developmental psychopathology

## Abstract

The present study aimed to investigate alexithymia and psychopathological manifestations centered on the body in a sample of adolescents with somatizing and/or self-harming issues to analyze the phenomenon of NSSI linked to Somatic Symptom Disorders (SSD). A sample of 184 adolescents between 12 and 19 years of age, was divided into three groups, one with NSSI (*n* = 49) and the second group with SSD (*n* = 57), comparing them with a third group of adolescents with SSD and NSSI (*n* = 78) to investigate their differences and similarities in psychopathological correlates and to analyze the mediation role of alexithymia and emotional dysregulation in NSSI and SSD related to internalizing problems. The battery of tests included the Toronto Alexithymia Scale (TAS-20) and the Youth Self-Report 11–18 (YSR). The NSSI + SSD group scored higher than the other two groups on all the YSR scales. The affective syndromes were the only clinical condition that discriminated between the three groups. On all the other syndrome scales, the NSSI + SSD group differed from the other two groups, while there were no differences between the NSSI group and the SSD group. The NSSI + SSD group revealed a more severely deficient emotional self-regulation. Difficulty identifying feelings was a trait shared by adolescents with SSD and those engaging in NSSI, a more complex overall alexithymia profile was associated with the combination of self-harming behavior and somatization. Alexithymia and emotional dysregulation played a mediating role in the relationship between internalizing problems and somatization. We did not find a mediating role in alexithymia and emotional dysregulation in the relationship between internalizing problems and self-injurious behavior. The combination of NSSI and SSD gave rise to more severe psychopathological correlates, clinical levels of alexithymia, and more severe deficient emotional self-regulation. Results of mediation role indicated a link between alexithymia, emotional dysregulation, and somatization.

## 1. Introduction

In recent years there has been a marked increase in the numbers of adolescents engaging in self-harming behavior, with or without suicidal intent. Data collected by the Italian Statistics Institute (ISTAT) from 1995 to 2017 indicate a decline in cases of suicide among adolescents (−14%), and an increase in self-harming phenomena in this age group. A study by Brunner et al. [1] found that more than one in every four (27.6%) adolescents in Europe (mean age 14 years) have engaged occasionally or repeatedly in self-harming behavior; in Italy, the figure stands at around 20%. Scientific, clinical, and social interest in self-harming phenomena has, consequently, also increased significantly in recent times. Efforts have focused on obtaining more information to help in its prevention, early diagnosis, and timely tailored treatment because individuals who engage in this type of behavior are considered at high risk of suicide [2,3,4,5]. Non-suicidal self-injury (NSSI) in adolescent age is seen as the end-result of a complex interaction between genetic, biological, psychiatric, psychological, social, and cultural factors. Many support the diathesis-stress model, according to which, a biological predisposition, personality, and cognitive vulnerabilities, associated with adverse events at an early age or in the recent past, and with psychiatric disorders, raise the lifetime risk of self-harming behavior [6]. Some of the psychological and intrapersonal aspects predictive of self-harming behavior reported in the literature include: a tendency for perfectionism; a limited problem-solving capacity; a self-critical style; alexithymic traits; and impulsiveness [5,7,8,9,10,11,12,13]. Emotional dysregulation also seems to be strongly associated with NSSI [14,15,16,17]. Much attention has also been paid in the literature to the psychopathological correlates associated with self-harming behavior. In particular, NSSI often appears to be associated with borderline personality disorder (BPD) [14] and with other psychiatric diagnoses of both internalizing and externalizing type—including mood disorders [1,6,17,18,19], eating disorders [18,20,21,22], and substance abuse [19]—especially in adolescence [6]. One interesting psychopathological correlate of self-harming that has been examined less often in the literature concerns symptoms of somatization. De Klerk et al. [15] identified routine self-harming and suicidal ideation in 45% of 461 psychiatric outpatients with somatoform disorders. Other studies showed that: people tended to contact health services before an episode of self-harming or attempted suicide, often complaining of physical problems rather than psychological issues [23,24]; more than one in two adolescents who attempted suicide had contacted a physician about physical symptoms in the previous week, without mentioning any thoughts of dying [25]; and the suicide rate among adolescents with functional disorders exceeded 30% [26]. The relationship between NSSI and Somatic Symptom Disorders (SSD) was recently investigated by Raffagnato et al. [27] in relation to any presence of alexithymia, which is also known to be associated with SSD [28,29] when an individual’s body expresses emotions they are unable to put into words. Based on empirical studies on the link between alexithymia and both NSSI and SSD, the authors suggest that the body is used as a way to cope with psychological suffering in both the latter conditions. Alexithymia emerged as a factor strongly associated with NSSI. On the other hand, SSD was found to be strongly associated with alexithymia (such that 95% of people presenting with somatic complaints were alexithymic as well, though just under half of alexithymic patients revealed SSD), but not necessarily with self-harming behavior. This could mean that alexithymia is a factor involved in the onset of both NSSI and SSD.

The global aim of the present work was to further analyze the phenomenon of NSSI in adolescence. A sample of adolescents engaging in self-harming behavior was divided into two groups, one with and the other without associated SSD, comparing them with a third group of patients with SSD without NSSI. The goals of the study were:

(a) To obtain information on the three groups’ psychopathological correlates in order to investigate their differences and similarities. Based on literature we expect differences between the three groups: in the NSSI + SSD group we expect more severe internalizing and externalizing psychopathology than in the other two groups, furthermore we assume differences between NSSI group and SSD group, in particular that NSSI correlate more with mood disorders [27,30] and SSD correlate more closely than NSSI with anxiety [31];
(b) To further investigate the mediation role of alexithymia and emotional dysregulation in NSSI and SSD related to internalizing problems. Our hypothesis emerging from the literature suggests that emotional dysregulation may play a mediating role in the development of self-harm, particularly in individuals with mood disorders, interpersonal problems, history of trauma, and physical and emotional abuse [14,32,33,34]. Furthermore, we hypothesise that alexithymia represents mediating factor in the development of self-harm in the context of internalizing problems, particularly in situations of exposure to traumatic experiences and bullying, according to Norman and Borrill [11].

## 2. Materials and Methods

The present study was part of a broader project on self-harming behavior approved by the ethical committee of Padua Hospital (CESU—Comitato Etico per la Sperimentazione Umana, n. 23—10.10.19). It was designed as a retrospective case-control study, based on a review of the data contained in the clinical files of patients aged from 12 to 19 years attending secondary- and tertiary-level pediatric neuropsychiatry services in an area of the Veneto region (north-east Italy). The adolescents selected for the study and their parents all gave their consent to their participation in the study (the neuropsychiatry services all adopt an approved official protocol that uses standardized forms regarding confidentiality and informed consent to data collection for clinical and research purposes).

The battery of tests routinely administered to patients includes the Toronto Alexithymia Scale (TAS-20) and the Youth Self-Report 11–18 (YSR), which were considered for the purposes of the present study.

The adolescents in each of three groups were selected on the grounds of several criteria. The sample of NSSI cases was divided into two groups: patients in the NSSI + SSD group had to have experienced at least one episode of self-harming, and to have scored at least as borderline cases of SSD (>60) on the DSM-oriented scale in the Youth Self-Report 11–18 (YSR); patients in the “NSSI” group had to have experienced at least one episode of self-harming behavior. For this latter group, the frequency of self-harming episodes (occasional or habitual, i.e., less or more than five episodes a year, respectively, according to the DSM-5criteria) was also considered. For inclusion in the third, a control group of adolescents with SSD (Somatic Symptom Disorders), these patients had to have at least borderline scores for SSD (>60) on the DSM-oriented scale in the Youth Self-Report 11–18 (YSR), and to have never engaged in self-harming behavior.

The study involved 184 adolescents: 28 males (15.2% of the sample) and 156 females (84.8%), who were between 12 and 19 years of age (mean = 15.6; SD = 1.37). The three groups obtained from this sample included: 49 adolescents in the NSSI group, 11 males (22.4% of the group) and 38 females (77.6%), mean age 15.6 years (SD = 1.39); 78 adolescents in the NSSI + SSD group, 7 males (9.0% of the group) and 71 females (91.0%), mean age 15.2 years (SD = 1.38); and 57 adolescents in the SSD group, 10 males (17.5% of the group) and 47 females (82.5%), mean age 16 years (SD = 1.32).

The Toronto Alexithymia Scale (TAS-20) [35] is a self-report questionnaire used to assess alexithymic traits. The latest version comprises 20 items investigating three dimensions of alexithymia: difficulty identifying feelings; difficulty describing feelings; and externally-oriented thinking. Respondents answer each item using a five-point Likert scale to indicate how much they agree with a set of proposed claims. Based on the sum of their scores, respondents may be identified as alexithymic (scores > 60), possibly alexithymic (scores between 51 and 60) or non-alexithymic (scores < 51). The scale also generates an alexithymic profile based on the total score obtained from the scores obtained in the three above-mentioned dimensions. 

The Youth Self-Report 11–18 (YSR 11–18) is self-report tool developed by Achenbach and Rescorla [36] (2001). It is one of the most widely-used scales for assessing young people’s behavior in Italy and internationally, in both clinical and research settings. The YSR is divided into two sections: the first investigates competences (activities, hobbies, sociality, and schooling) with 20 items; the second investigates emotional and behavioral issues with 118 items. The YSR generates two profiles: one for competences, consisting of three scales (activities, sociality, and academic performance); and a psychological and/or psychopathological profile that refers to eight syndrome scales, anxiety/depression, withdrawal, somatic complaints, social problems, thought problems, attention problems, antisocial behavior, and aggressive behavior. These syndrome scales are grouped into three problem scales: internalizing problems (anxiety/depression, withdrawal, and somatic complaints); externalizing problems (antisocial behavior and aggressive behavior); and other, neither internalizing nor externalizing, problems (social problems, thought problems, and attention problems). A total problems score is also calculated. The scores on the various scales for behavioral and emotional problems are quantified in the range of “normal”, “borderline” or “clinical” in relation to normative values for a given age group. There are also six DSM-oriented scales (DOS) relating to: Affective Problems, Anxiety Problems, Somatic Problems, Attention Deficit/Hyperactivity Problems, Oppositional Defiant Problems, and Conduct Problems. Based on the scores they obtained on the syndrome scales for anxiety/depression, attention problems, and aggressive behavior, individuals’ emotional-behavioral regulation was classified as: no dysregulation for T scores < 180; a moderately deficient emotion self-regulation for T scores from 180 to 210; or a full-blown dysregulation profile for T scores ≥ 210 [37,38,39,40,41].

The data were analyzed using the “Jamovi” statistical software (a free and open statistical platform; The jamovi project (2021). *jamovi* (Version 1.6) [Computer Software]. Retrieved from https://www.jamovi.org accessed on 6 February 2022; Sydney, Australia). To answer our research questions, the data were processed using descriptive statistics of the scores obtained in each test by group. Univariate analysis of variance (ANOVA) and the Games-Howell post-hoc test were used to pinpoint differences or similarities between the three groups’ psychopathological characteristics and alexithymic profiles. A bivariate parametric correlation analysis (Pearson’s r coefficient) was used to identify correlations between the clinical characteristics and the groups. Qualitative variables were investigated using the chi-squared test. To test Alessitimia and emotional dysregulation as mediators between internalizing problems, and NSSI and SSD, we compute a GLM Mediation Analysis. The statistical analyses considered in the study were two-way and the threshold for statistical significance was set at *p* < 0.05. MANCOVA was used to analyze the differences in the psychopathological and alexithymia profiles by controlling for gender.

## 3. Results

### 3.1. Psychopathological Characteristics

Our data were processed using descriptive statistics to investigate the differences between the three groups’ psychopathological profiles, as assessed with the YSR 11–18 (Table 1).

Differences in the three groups’ psychopathological profiles were analyzed using ANOVA Statistically significant differences emerged between the three groups for all the scales investigated except for “Activities” on the Competences scale.

The Games Howell post-hoc test was used to see which groups were affected by the differences that gave rise to the overall significance on the syndrome scales of the YSR 11–18. From these analyses, statistically significant differences between the SSD group and the NSSI + SSD group only emerged on the subscales concerning: Competences (for Social competences, *p* = 0.006; and Total competences, *p* = 0.030); and Attention (Attention problems, *p* < 0.001; and Attention deficit/hyperactivity, *p* < 0.001). On the other hand, all three groups differed on the scales for Affective problems, which differentiated between the NSSI and the NSSI + SSD groups (*p* < 0.001), between the NSSI and the SSD groups (*p* = 0.005), and between the NSSI + SSD and the SSD groups (*p* < 0.001). On all the other syndrome scales, the NSSI + SSD group differed from the other two groups, while there were no differences between the NSSI group and the SSD group (Table 2). In short, the affective syndromes were the only clinical condition that discriminated between the three groups.

For most of the syndrome subscales, the NSSI + SSD group scored the highest. More in detail, when our data were analyzed with the chi-squared test, 28% of the SSD group, 37% of the NSSI group, and 54% of the NSSI + SSD group obtained clinically significant scores on the scale for externalizing problems (χ^2^ = 16.0, *p* < 0.001). Concerning the scale for internalizing problems, the clinical cutoff was exceeded by 68% of the adolescents in the SSD group, 71% in the NSSI group, and 94% in the NSSI + SSD group (χ^2^ = 9.60, *p* = 0.008). On the affective problems scale, clinical scores were obtained by only 35% of the SSD group, 57% of the NSSI group, and 86% of the NSSI + SSD group (χ^2^ = 37.1, *p* < 0.001).

### 3.2. Impairments in Emotion Regulation

The levels of emotional-behavioral dysregulation in the three study groups were investigated in terms of the emotional self-regulation profile derived from the YSR syndrome scales for anxiety/depression, attention problems, and aggressiveness. The results of our ANOVA showed statistically significant differences between the three groups’ dysregulation profiles (F = 18.3; *p* < 0.001): the NSSI + SSD group revealed a more severely deficient emotional self-regulation (M = 200; SD = 24.1) than the NSSI group (M = 182; SD = 19.1) or the SSD group (M = 178; SD = 20.0). The Games Howell Post-Hoc test identified no differences between the NSSI group and the SSD group, confirming that it was the group with both NSSI and SSD that differed from the other two (NSSI, *p* < 0.001; SSD, *p* < 0.001). When the cutoffs were analyzed, a severe dysregulation profile (≥210) was only seen in a few individuals in the SSD group (9%) and the NSSI group (10%), whereas this cutoff was exceeded much more often in the NSSI + SSD group (40%). A moderately deficient emotional self-regulation (T scores from 180 to 210) was identified in 26% of the SSD group, 41% of the NSSI group, and 37% of the NSSI + SSD group (χ^2^ = 34.4, *p* < 0.001). To be specific, clinical scores on the aggressive behavior scale were obtained for about 18% of adolescents in the SSD group, 12% in the NSSI group, and 35% in the NSSI + SSD group (χ^2^ = 9.97, *p* = 0.007). The situation was similar for attention problems, with scores in the clinical range for by 21% of the SSD group, 31% of the NSSI group, and 46% of the NSSI + SSD group (χ^2^ = 9.61, *p* = 0.008). On the scale for anxiety/depression, clinically relevant scores were recorded for 44% of the SSD group, 59% of the NSSI group, and 78% of the NSSI + SSD group (χ^2^ = 16.9, *p* < 0.001). In all three groups, therefore, the scale for anxiety/depression was the most representative of the scales contributing to the dysregulation profile. The NSSI + SSD group differed from the other two groups in that it had a more severe profile of emotional-behavioral dysregulation, as revealed, in particular, by higher clinical scores than in the other two groups on the scales for anxiety/depression and externalizing problems.

### 3.3. Alexithymia in the Three Groups

An ANOVA was run to identify any differences in the alexithymia profiles of our study groups. The results showed statistically significant differences between the three groups, in the three factors investigated with the TAS 20 and in the total TAS 20 scores: difficulty identifying feelings (F = 9.03, *p* < 0.001); difficulty describing feelings (F = 10.35, *p* < 0.001); externally-oriented thinking (F = 11.29, *p* < 0.001); and total for alexithymia (F = 16.52, *p* < 0.001). Table 3 shows the descriptive statistics for the scores obtained on the TAS-20 by the three study groups.

When the Games Howell post-hoc test was used to see which differences between the groups gave rise to the statistical significance, the pattern regarding their alexithymia profiles was much the same as the one that emerged for the sample’s psychopathological characteristics. There were statistically significant differences in terms of the adolescents’ difficulty identifying feelings, difficulty describing feelings, and total scores for alexithymia, between the SSD group and the NSSI + SSD group (*p* < 0.001; *p* = 0.001; *p* < 0.001) and between the NSSI group and the NSSI + SSD group (*p* = 0.008; *p* < 0.001; *p* < 0.001). For externally-oriented thinking, on the other hand, the only statistically significant difference was between the SSD group and the NSSI + SSD group (*p* < 0.001). Once again, therefore, the SSD group did not differ to any statistically significant degree from the NSSI group.

In descriptive terms, only 39% of the SSD group, and 45% of the NSSI group obtained clinically relevant scores, whereas the NSSI + SSD group scored in the clinical range in 74% of cases (χ^2^ = 19.4, *p* < 0.001). In short, alexithymia was more common in adolescents with both NSSI and SSD. Pearson’s correlation analyses between the three groups’ scores on the syndrome scale for Somatic complaints and on the TAS 20 were analyzed to shed more light on the relationship between alexithymia and somatization. The result re-elevated the statistically significant correlations between somatic complaints and difficulty identifying feelings in SSD (r = 0.342; *p* = 0.009) and NSSI groups (r = 0.312; *p* = 0.033) while in the NSSI + SSD groups the somatic complaints correlated with difficulty identifying feelings (r = 0.328; *p* = 0.004), difficulty describing feelings (r = 0.250; *p* = 0.032) and with total for alexithymia (r = 0.346; *p* = 0.003).

In the SSD group and the NSSI group, the scores for Somatic complaints only correlated with those on the TAS 20 subscale regarding difficulty identifying feelings, whereas the NSSI + SSD group’s scores for Somatic complaints correlated significantly with their scores on three of the TAS-20 subscales (identifying feelings, describing feelings, and total for alexithymia). We analyzed differences in the psychopathological and alexithymia profiles profile with MANCOVA by controlling for gender but found no statistically significant differences.

### 3.4. Mediation Role of Alexithymia and Emotional Dysregulation

**Figure 1:**
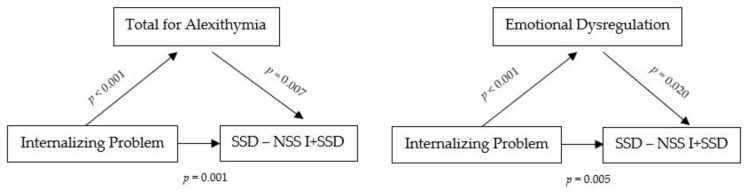
Mediation role of alexithymia and emotional dysregulation.

In order to investigate alexithymia and emotional dysregulation as mediators between internalizing problems and NSSI and SSD, we computed a GLM Mediation Analysis in parallel. Figure 1 and Table 4 show these GLM results; emotional dysregulation plays a role in mediating the effects of individual impact of the internalizing problems and somatization (β = 0.166, SE = 0.003, Bootstrap 95% CI [0.001, 0.015], *p* = 9.023; R^2^ = 0.24). Regarding the role of alexithymia, the construct totally mediates the relationship between internalizing problems and somatization (β = 0.146, SE = 0.003, Bootstrap 95% CI [0.002, 0.012], *p* = 0.010; R^2^ = 0.25).

GML analysis in parallel was conducted to examine the mediating role of alexithymia and emotional dysregulation in the relationship between internalizing problems and self-harming behavior. The results did not show a mediating role (Alexithymia: β = 0.045, SE = 0.003, Bootstrap 95% CI [−0.004, 0.008], *p* = 0.494; Emotional dysregulation: β = 0.048, SE = 0.003, Bootstrap 95% CI [−0.004, 0.008], *p* = 0.483).

## 4. Discussion

The present study aimed to investigate alexithymia and psychopathological manifestations centered on the body in a sample of adolescents with somatizing and/or self-harming issues. There was a marked prevalence of females in our sample (84.8%); this is consistent with other reports in the literature, which identifies females as being at higher risk of these clinical conditions in adolescence, in the absence of comorbidities such as substance abuse [6,15,18,42,43,44].

The first aim of our study was to identify any differences and/or similarities in the clinical and psychopathological characteristics of our groups of adolescents with NSSI or SSD, issues, or both. While no statistically significant differences emerged between the SSD group and the NSSI group on the majority of the scales investigated, the NSSI + SSD group scored higher than the other two groups on all the YSR scales. This would suggest that the combination of the two disorders gives rise to more severe psychopathological correlates. It is particularly worth mentioning that the three groups differed in terms of affective problems: more than half of the NSSI group had clinical scores on this subscale, and almost all of the adolescents in the NSSI + SSD group exceeded the clinical cutoff. A correlation between NSSI and affective disorders was identified in several published studies [1,14,18,27,45,46] and several authors have suggested that mood disorders might predict the onset of self-harming behavior [30].

Based on our hypotheses, our results show that self-harming behavior did correlate with mood disorders more than with somatization [27,30]. In contrast, no differences emerged between the SSD group and the NSSI group as concerns the scale for anxiety.

Generally speaking, internalizing problems were more representative of the clinical situation than externalizing problems in our sample. Together with emotional-behavioural dysregulation, externalizing problems were a distinctive feature of the NSSI + SSD group, as they were found in a larger percentage of this group than in the groups with SSD or NSSI alone. Concerning their capacity for emotion regulation, the adolescents in our NSSI + SSD group often showed clinical signs of dysregulation according to the YSR scales (a dysregulation profile and deficient emotional self-regulation), and the more severe cases were in the NSSI + SSD group. In the literature, emotion regulation has often been studied as a factor with a possible role in self-harming behavior [46]. Individuals with a deficient emotional self-regulation would engage in NSSI as a dysfunctional strategy for managing their emotions, as it reduces the experience of negative affects and/or serves as a distraction from them [15,17,46]. In a study by Glenn and Klonsky [20], self-harming patients obtained high scores on the Difficulties in Emotion Regulation Scale [16], and higher levels of emotional dysregulation were associated with a greater frequency of NSSI.

On the other hand, few studies conducted to date have investigated the relationship between somatization and emotional dysregulation. Research on patients with somatoform disorders suggests that they focus on, amplify, and misinterpret the bodily sensations that accompany their emotions due to a limited capacity to use cognitive mechanisms to understand and regulate them [47]. According to Martin and Pihl [48], failure to regulate and modulate emotions in times of stress can engender exaggerated physiological and behavioral responses and a greater vulnerability to somatic disorders [49]. In our study sample, more than half (about 65%) of the patients with SSD did not reveal problems with emotion regulation, which would suggest that emotion regulation is related more closely to self-harming behavior than to somatic symptoms. This would be consistent with the assumption that the emotional dysregulation profile—known as the A-A-A profile because it is calculated from the scores obtained on the anxiety/depression, attention problems, and aggressive behavior scales of the YSR—reflects a type of dysregulation with a mainly externalizing expression. On the other hand, all three groups in our sample had higher clinical scores on the scale for anxiety/depression than on the scales for attention problems or aggressive behavior, confirming a preponderance of affective problems.

The present study also tried to further analyse the relationship between alexithymia and psychopathological conditions like SSD and NSSI that are centered on the body. Various studies found that adolescents engaging in self-harming behavior struggled more with identifying and describing feelings [10,50,51], just as SSD has been associated with greater difficulty in identifying and describing feelings [49]. Alexithymia is a factor common to SSD and NSSI: individuals who tend to somatize and those who engage in self-harming behavior both use their own body to manage a psychological and emotional suffering that they are unable to put into words [52].

An analysis was conducted on the relationship between alexithymia and somatic complaints in adolescents with and without associated self-harming behavior. It emerged that, while difficulty identifying feelings was a trait shared by adolescents with SSD and those engaging in NSSI, a more complex overall alexithymia profile (difficulty identifying feelings, difficulty describing feelings, and externally-oriented thinking) was associated with the combination of self-harming behavior and somatization. In accordance with the second hypothesis of the study, to explain the mediation role of alexithymia and emotional dysregulation in NSSI and SSD related to internalizing problems, we conducted further analysis. GML results showed that alexithymia and emotional dysregulation plays a mediating role in the relationship between internalizing problems and somatization. These results indicate a link between alexithymia, emotional dysregulation and somatization. On the other hand, we did not find a mediating role in alexithymia and emotional dysregulation in the relationship between internalizing problems and self-injurious behavior in contrast to some studies in the literature that highlight the mediating role of emotional dysregulation in the engagement of NSSI, especially in individuals with mood disorders, history of trauma, physical and emotional abuse [33,34] and the mediating role of alexithymia in the context of internalizing problems, particularly in situations of exposure to traumatic experiences and bullying episodes [11]. This probably points to a limitation in our NSSI classification that includes individuals who reported fewer than five episodes of self-harm and highlights the need to incorporate more indicators, including history of abuse and trauma, in order to better explain the relationship between these variables.

## 5. Conclusions

Despite this study’s strengths and originality, some limitations need to be taken into account. For a start, the sample was numerically small. Second, the use of self-report questionnaires can be susceptible to social desirability bias. The use of self-report questionnaires to assess alexithymia—which demands a certain capacity for introspection—could also be negatively influenced by the psychocognitive characteristics of alexithymic individuals. A final weakness is not having a control group from the normative population to make a further comparison.

Albeit, with these limitations, our findings can provide useful theoretical-clinical input, and entitle us to claim that a combination of alexithymia and somatization in self-harming patients is associated with more severe clinical picture. Investigating these aspects in patients who engage in self-harming, both clinically and with the aid of tests—and possibly taking into account the environment in which patients live and the dynamics of their family interactions [45,53,54]—could help us to identify the individuals at greater psychopathological risk. This is an interesting finding from a preventive and intervention perspective, suggesting the importance of work on emotional awareness, on the capacity to recognize and express feelings to acquire competence in their regulation.

## Figures and Tables

**Table 1:** Mean scores obtained by the three groups on the Youth Self-Report 11–18 (YSR) scales and differences between the three groups’ psychological profiles on univariate ANOVA.

		YSR			
		SSD	NSSI	NSSI + SSD	ANOVA
Activities	M(SD)	40.6 (10.28)	36.3 (9.12)	38.1 (9.55)	F = 2.51; *p* = 0.086
Social	M(SD)	44.2 (9.87)	39.5 (11.20)	39.0 (8.77)	F = 5.22; *p* = 0.007
Total competences	M(SD)	39.6 (9.42)	34.7 (11.01)	35.3 (8.88)	F = 4.11; *p* = 0.019
Internalizing Problems	M(SD)	64.2 (8.61)	63.3 (8.97)	73.8 (9.78)	F = 25.26; *p* < 0.001
Externalizing Problems	M(SD)	55.2 (8.48)	55.1 (8.63)	60.9 (9.66)	F = 8.53; *p* < 0.001
Total Problems	M(SD)	60.5 (7.69)	60.7 (8.21)	69.7 (8.26)	F = 28.34; *p* < 0.001
Anxious/Depressed	M(SD)	63.4 (9.44)	64.9 (8.79)	73.8 (12.98)	F = 16.11; *p* < 0.001
Withdrawn/Depressed	M(SD)	61.1 (10.60)	65.5 (10.36)	72.0 (12.65)	F = 14.63; *p* < 0.001
Somatic Complaints	M(SD)	64.2 (6.49)	55.5 (4.19)	69.2 (7.46)	F = 96.00; *p* < 0.001
Social Problems	M(SD)	59.1 (10.38)	62.9 (9.08)	67.9 (9.60)	F = 12.97; *p* < 0.001
Thought Problems	M(SD)	57.2 (6.13)	60.2 (7.94)	67.4 (10.56)	F = 25.09; *p* < 0.001
Attention Problems	M(SD)	57.6 (9.24)	61.4 (9.09)	64.7 (9.83)	F = 9.20; *p* < 0.001
Rule-Breaking Behavior	M(SD)	56.5 (6.57)	56.8 (7.46)	60.8 (8.66)	F = 6.04; *p* = 0.003
Aggressive Behavior	M(SD)	56.6 (6.87)	56.2 (6.42)	61.2 (9.14)	F = 7.71; *p* < 0.001
Affective Problems	M(SD)	60.3 (7.90)	66.2 (10.53)	75.8 (11.37)	F = 43.31; *p* < 0.001
Anxiety Problems	M(SD)	61.1 (7.39)	61.3 (7.94)	66.3 (8.44)	F = 9.01; *p* < 0.001
Somatic Problems	M(SD)	65.0 (6.18)	52.9 (2.62)	68.4 (7.48)	F = 196.95; *p* < 0.001
Attention/Deficit	M(SD)	54.8 (5.41)	56.7 (5.88)	58.9 (7.10)	F =7.55; *p* < 0.001
Oppositional Defiant Problems	M(SD)	55.7 (6.59)	56.4 (6.14)	60.0 (8.18)	F = 6.39; *p* = 0.002
Conduct Problems	M(SD)	53.3 (7.13)	54.9 (6.84)	60.0 (9.20)	F = 12.03; *p* < 0.001

**Table 2:** Games Howell post-hoc test with statistically significant differences between the NSSI + SSD group and the other two groups on the Youth Self-Report 11–18 (YSR) scales.

NSSI + SSD		NSSI	SSD
Internalizing problems	*p*-value	<0.001	<0.001
Externalizing problems	*p*-value	0.002	0.001
Total problems	*p*-value	<0.001	<0.001
Anxiety/depression	*p*-value	<0.001	<0.001
Withdrawal/depression	*p*-value	0.007	<0.001
Social problems	*p*-value	0.011	<0.001
Thought problems	*p*-value	<0.001	<0.001
Rule-breaking behavior	*p*-value	0.018	0.004
Aggressive behavior	*p*-value	0.001	0.003
Anxiety problems	*p*-value	0.003	<0.001
Oppositional defiant problems	*p*-value	0.017	0.003
Conduct problems	*p*-value	0.002	<0.001

**Table 3:** Mean scores obtained by the three study groups on the Toronto Alexithymia Scale (TAS-20) scales.

TAS-20		SSD	NSSI	NSSI + SSD
Difficulty identifying feelings	M(SD)	20.2 (5.91)	21.0 (6.26)	24.6 (6.45)
Difficulty describing feelings	M(SD)	15.9 (4.73)	15.4 (4.90)	18.7 (4.03)
Externally-oriented thinking	M(SD)	20.3 (3.49)	21.9 (4.35)	23.6 (4.59)
Total for alexithymia	M(SD)	56.5 (10.74)	58.3 (11.84)	66.9 (11.06)

**Table 4:** GLM Mediation Analysis.

Type	Effect	Estimate	SE	95% C.I.		β	z	*p*
				Lower	Upper			
Indirect	Internalizing Problems ⟹ Total for Alexithymia ⟹ SSD-NSSI + SSD	0.007	0.003	0.002	0.012	0.146	2.56	0.010
Component	Internalizing Problems ⟹ Total for Alexithymia	0.677	0.083	0.515	0.840	0.581	8.16	<0.001
	Total for Alexithymia ⟹ SSD-NSSI + SSD	0.010	0.004	0.003	0.018	0.251	2.70	0.007
Direct	Internalizing Problems ⟹ SSD-NSSI + SSD	0.015	0.004	0.006	0.024	0.306	3.28	0.001
Total	Internalizing Problems ⟹ SSD-NSSI + SSD	0.022	0.004	0.014	0.029	0.452	5.77	<0.001
Indirect	Internalizing Problems ⟹ Emotional Dysregulation ⟹ SSD-NSSI + SSD	0.008	0.003	0.001	0.015	0.166	2.28	0.023
Component	Internalizing Problems ⟹ Emotional Dysregulation	1.647	0.150	1.353	1.940	0.687	10.99	<0.001
	Emotional Dysregulation ⟹ SSD-NSSI + SSD	0.005	0.002	7.61 × 10^−4^	0.009	0.241	2.33	0.020
Direct	Internalizing Problems ⟹ SSD-NSSI + SSD	0.014	0.005	0.004	0.023	0.290	2.80	0.005
Total	Internalizing Problems ⟹ SSD-NSSI + SSD	0.022	0.004	0.015	0.029	0.455	5.92	<0.001

Note. Confidence intervals computed with method: Standard (Delta method). Betas are completely standardized effect sizes.

## Data Availability

Restrictions apply to the availability of these data. Data were obtained from a broader project about self-harming behavior by approved by Institutional Ethical Commission and are available from the Principal Investigation (MG) on reasonable request.
